# ApoM binds endotoxin contributing to neutralization and clearance by High Density Lipoprotein

**DOI:** 10.1016/j.bbrep.2023.101445

**Published:** 2023-02-28

**Authors:** Hanaa Mousa, Angelos Thanassoulas, Susu M. Zughaier

**Affiliations:** Department of Basic Medical Sciences, College of Medicine, QU Health, Qatar University, Doha, P.O. Box 2713, Qatar

**Keywords:** ApoM, Endotoxin, Isothermal calorimetry, Lipopolysaccharide, HDL, Neutralization, TNFα

## Abstract

**Background:**

HDL possesses anti-inflammatory properties, however, the exact mechanism is not fully understood. Endotoxin is a potent inducers of TLR4 signaling, leading to inflammatory mediators’ release. It has been estimated that TLR4 recognizes about 5% of circulating lipopolysaccharide whereas 95% is cleared by plasma lipoproteins, mainly HDL. ApoM is required for HDL biogenesis and 95% of plasma ApoM is found associated with HDL, both are significantly reduced during sepsis.

**Aim:**

The aim of this study is to investigate whether ApoM binds endotoxin and contributes to anti-inflammatory activity of HDL.

**Methods:**

Isothermal Titration Calorimetry (ITC) was used to determine the binding of ultrapure *E. coli* LPS to the recombinant ApoM protein. Purified human HDL and recombinant ApoM was used to investigate LPS neutralization using human and murine macrophages and computational simulation was performed.

**Result:**

ApoM shows high affinity for *E. coli* LPS, forming 1:1 complexes with Kd values below 1 μΜ, as revealed by ITC. The binding process is strongly exothermic and enthalpy-driven (Δ_r_H = −36.5 kJ/mol), implying the formation of an extensive network of interactions between ApoM and LPS in the bound state. Computational simulation also predicted high-affinity binding between ApoM and *E. coli* LPS and the best scoring models showed *E. coli* LPS docking near the calyx of ApoM without blocking the pocket. The biological significance of this interaction was further demonstrated in macrophages where purified HDL neutralized an *E. coli* LPS effect and significantly reduced TNFα release from human THP-1 cells.

**Conclusion:**

ApoM binds LPS to facilitate endotoxin neutralization and clearance by HDL.

## Highlights

• HDL neutralizes endotoxins and reduces TNFα release.
• ApoM binds with high affinity to *E. coli* LPS as demonstrated by isothermal titration calorimetry.
• Recombinant ApoM reduced endotoxin immune stimulatory activity.
• Computational modelling predicted that LPS binds to ApoM surface near calyx but does not block S1P binding pocket.

## 1 Introduction

Sepsis is a life threating emergency resulting in high mortality and morbidity around the world. Around 20% of deaths are sepsis-related [1], which is caused by an exacerbated immunological reaction towards bacterial endotoxins or infection, leading to organ failure and death [2]. Endotoxin also known as Lipopolysaccharide (LPS) is a major component of the outer membrane of gram-negative bacteria, such as *E. coli, Salmonella, and Neisseria meningitidis* [3]. LPS activates the immune response via toll-like receptor 4 (TLR4) by binding to its co-receptor MD-2. TLR4 is a transmembrane glycoprotein highly expressed on the surface of immune cells, particularly macrophages [4]. TLR4 recognizes about 5% of circulating endotoxin/LPS [5]. The binding of LPS in the calyx of MD-2 leads to TLR4 dimerization and initiation of signaling cascade leading to pathways activation such as NF-κB complex (nuclear factor kappa-light-chain-enhancer of activated B cells), and releases some inflammation and cytokines mediators e.g. TNFα [[6], [7], [8]].

The clearance of endotoxin from circulation is mediated by HDL [[9], [10], [11], [12]], which is known as the good cholesterol owing to its role in reverse cholesterol transport and cardiovascular protection properties [13]. HDL belongs to the lipoproteins family and is composed of higher protein constituents relative to other members (e.g., LDL, VLDL, triglycerides) [14]. HDL main function is to transfer cholesterol from peripheral tissues to the liver where it is recycled or excreted as bile salts. This process is known as reverse cholesterol transport (RCT). The HDL associated apolipoproteins interact with numerous cellular receptors which enables the cholesterol efflux from cells to the HDL particle [15]. Moreover, apolipoproteins associated with HDL particles possess other functions like anti-inflammatory or antioxidative properties [16]. As a result, HDL particles undergo continuous remodeling in physical structure and constituents [17]. Importantly, inflammation disrupts the RCT pathway by reducing cholesterol trafficking from macrophage foam cells to the liver, leading to dysfunctional HDL particles [18].

A reduction in HDL was observed during sepsis, suggesting a role of HDL in containing and suppressing the exacerbated immunological response [19,20]. Studies have ascribed this decrease in HDL to the disturbance or dysfunction of the lipoproteins constituents leading to reduced HDL biogenesis [21,22]. ApoA-1, the major protein associated with HDL is shown to bind to endotoxin potentially aiding in clearance from circulation [23].

ApoM is one of the HDL associated apolipoproteins crucial for HDL biogenesis, and may contribute to the anti-inflammatory function of HDL. Around 95% of plasma ApoM is found associated with HDL [24]. ApoM is a 25 kDa protein, produced mainly in liver and kidney belongs to the lipocalin family (a group of proteins that transport small hydrophobic molecules) [25]. Structurally, ApoM has a typical lipocalin fold that consists of eight β-strands forming a barrel [26]. ApoM is a lipid carrier and documented to shuttle the bioactive sphingosine 1 phosphate (SIP), where ApoM-S1P axis plays critical role in modulating various diseases [24]. Similar to HDL, ApoM levels were also shown to be reduced during sepsis and severe inflammation [27,28]. In this study, we examined the role of HDL and ApoM in endotoxin neutralization and provide evidence that ApoM binds to LPS to facilitate its clearance, consequently contributing to the anti-inflammatory effects HDL.

## 2 Materials and methods

### 2.1 Reagents

Human TNFα DuSet ELISA kit (R&D Systems, Catalog Number: DY210), recombinant human ApoM protein (R&D Systems, Catalog Number: 4550-AM-050) and recombinant human LL-37 (TOCRIS, Catalog Number: 5213). Highly purified endotoxins *Salmonella typhimurium*, *E. coli B55*, *Salmonella minnesota* and *Vibrio cholerae* were described previously [29] and new lots of endotoxins were purchased from Invivogen; SM Ultrapure (Invivogen, Catalog Number: tlr1-sm lps) and LPS-B5 Ultrapure (Invivogen, Catalog Number: tlrl-pb5lps). Commercial HDL derived from human plasma was purchased from (Sigma-Aldrich, Catalog Number: L8039. St. Louis, USA). Pooled human plasma HDL and LDL fractions were a kind gift from Dr Ngoc-Anh Le (Atlanta Veterans Affairs Medical Center, Decatur, GA; and Emory University School of Medicine, Atlanta, GA, USA).

### 2.2 Endotoxin neutralization studies by HDL and LDL

Human THP-1 cells were cultured using RPMI 1640 supplemented with 5% penicillin streptomycin and 10% Fetal serum bovine. Freshly grown THP-1 cells were adjusted to 1 × 10^6^ cell/ml and 250 μl aliquots were transferred into 96-well plate then induced with 1 ng/ml of LPS derived from *Salmonella typhimurium*, *E. coli B55*, *Salmonella minnesota* and *Vibrio cholerae* with or without 10 μl of purified HDL, LDL (stock solution at 1 mg/ml) or PBS as control. The treated cells and controls were then incubated overnight at 37 °C with 5% CO_2_. Experiments were optimized in 96-well plate to mimic physiological conditions, hence the selected concentration of HDL. The released TNFα was measured in the supernatants using ELISA method following manufacturer instructions as previously described [29]. Briefly, TNFα capture antibody (R & D Systems, MN, USA) diluted in PBS was coated in 96-well plate overnight, followed by 1% BSA blocking buffer. Subsequently, 100 μl of supernatants from induced monocytic cells were added to the washed coated plates and incubated for 2 h at room temperature. After washing, the TNFα detection antibody was added and after 2 h, the substrate was introduced and developed following the manufacturer instructions. The fluorescence emission of the sample was monitored at 540 nm after excitation at 450 nm. The concentrations of TNFα were generated from the calculated standard curve using recombinant TNFα as previously described [29].

### 2.3 Isothermal titration calorimetry

A Nano ITC (TA Instruments) low volume isothermal titration calorimeter was employed to study the interaction between human ApoM and LPS from *E. coli*. All solutions used for ITC experiments were dissolved directly in the same batch of PBS buffer, to avoid buffer mismatch dilution heats. All samples were thoroughly degassed before use to avoid bubble formation. In a typical experiment, the calorimetric cell was filled with 10 μM of ApoM and the syringe was loaded with a 100 μM LPS solution. The titration sequence consisted of an initial 0.9 μL injection, followed by 15 identical 2.54 μL injections at 300s intervals. Experiments were performed at 25 °C and a stirring speed of 150 rpm was used to ensure a rapid equilibration of the mixture. Heat contribution from injectant dilution was accounted for in a separate experiment, by injecting LPS in buffer solution following an identical titration protocol. These dilution effects were subsequently subtracted from the titration data to obtain the net binding isotherm, as a function of the overall LPS concentration in the cell. All ITC data were processed using the NanoAnalyze software (TA Instruments, New Castle, DE, USA).

Complex formation is an equilibrium interaction that can be described by a chemical equation of the form:

$$\left[ApoM\right]+\left[LPS\right]K_{b}\;\left[Complex\right]$$

where: [*ApoM*] and [*LPS*] are the concentrations of the non-complexed ApoM and LPS respectively, [Complex] represents the concentration of the ApoM-LPS complex, while K_b_ = 1/*K*_d_ is the binding constant of the interaction.

The stoichiometry (moles of LPS bound per mol of ApoM) [N], the binding constant [K_b_] and the binding enthalpy [Δ_r_H] of the reaction are obtained, along with their corresponding uncertainties, directly from fitting the ITC experimental data to a one set-of-sites binding model. The Gibbs free energy change (Δ_r_G) and the entropy change (Δ_r_S) of the complexation are then calculated from the equations: Δ_r_G = RT ln K_b_ = Δ_r_H – T Δ_r_S, where R is the gas constant and T is absolute temperature. The uncertainties of these parameters were estimated using error propagation calculations.

### 2.4 Molecular docking simulations of ApoM - *E. coli* LPS binding

All computational procedures were carried out with the Molecular Operating Environment (MOE 2019.01, Chemical Computing Group, Montreal, Canada) software, using the Amber12: EHT force field with the reaction field electrostatics treatment. ApoM was treated as rigid for the docking simulations while conformational space was sampled for the LPS ligand (Template: PDB entry 2WEW). Briefly, 20.000 ligand conformations were generated by sampling their rotatable bonds and placed using the Triangle Matcher Method. Duplicate complex structures are then filtered out and the best 1.000 poses were scored according to the London dG empirical scoring function for an estimation of their binding energy [30]. The 100 top scoring complexes were submitted to a more in-depth refinement step based on molecular mechanics and the structures produced were re-evaluated using the GBVI/WSA ΔG empirical scoring function to include solvation effects [31]. Ten or less structures are generated at this stage. Finally, the MOE 2019 LigX script was applied to the best pose to minimize the energies of both the ligand and the receptor, in order to get a more accurate estimation of the ligand affinity.

### 2.5 Endotoxin neutralization by recombinant ApoM

*E. coli* LPS with different concentrations of 100 and 50 ng/ml were pre-incubated with recombinant ApoM protein (10 μg/ml) in a 96-well plate for 30 min. Freshly grown murine RAW264 macrophages where then added to 96-well plate at 0.25 × 10^6^ cell/well and further incubated overnight. Nitric oxide release was measured as nitrite accumulation using the Greiss reaction as previously described [29]. Recombinant LL-37 is a host cationic peptide also known as cathelicidin was used as a positive control due to its ability to neutralize LPS immune-stimulatory activity [32].

### 2.6 Statistical analysis

Graphpad Prism 5 was used in analysis and generating figures, T-test was calculated to compare HDL and LDL in relation to PBS (Cells treated with PBS considered as a control). *P* value < 0.05 considered significant.

## 3 Results

### 3.1 HDL neutralized endotoxin and decreased TNF-α release from human THP-1 cells

**Fig. 1:**
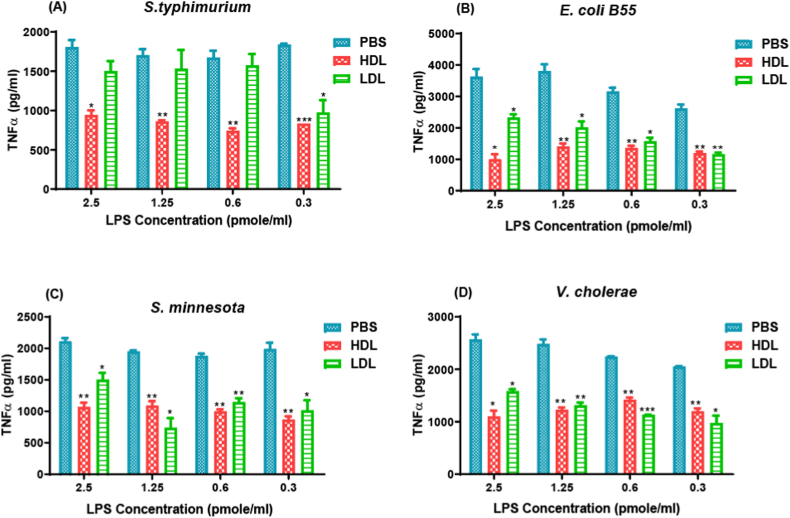
Endotoxin neutralization by HDL decreased TNF-α release from human THP-1 cells. Endotoxin doses ranging between 2.5 and 0.3 pmol/ml were pre-incubated with purified HDL or LDL fractions (10 μg/ml) or PBS for 30 min prior to addition to THP-1 cells (0.25 × 10^6^/ml) and further overnight incubation at 37 °C. TNF-α release from induced THP-1 cells was measured using ELISA method. Endotoxins used were *S. typhimurium* (**A**), *E. coli B55* (**B**), *S. minnesota* (**C**) and *V. cholera* (**D**). *P value < 0.05, **P value < 0.01 and ***P value < 0.001.

The ability of purified HDL fraction to neutralize endotoxin was examined *in vitro.* TNF-α secretion from human THP-1 cells induced with various endotoxins in presence and absence of HDL was measured. HDL (10 μg/ml) pre-incubation with various doses of endotoxins (2.5–0.3 pmol/ml) purified from *S. typhimurium*, *E. coli B55*, *S. minnesota* and *V. cholera* prior to addition to THP-1 cells resulted in a significant reduction in TNF-α release (Fig. 1).

Endotoxin concentrations used here are very low and thereby physiologically relevant. HDL neutralized endotoxins more effectively compared to LDL fraction and reduced TNF- α release especially at higher doses of endotoxins *S. typhimurium* (Fig. 1A), *E. coli B55* (Fig. 1B), *S. minnesota* (Fig. 1C), and *V. cholerae* (Fig. 1D). However, effective endotoxin neutralization by both HDL and LDL fractions was observed at lower doses of endotoxins.

### 3.2 Computational docking simulations of ApoM - *E. coli* LPS binding

ApoM, a lipid carrier protein, is critical for HDL biogenesis. Studies documented that both HDL and ApoM are reduced during sepsis. We examined our hypothesis that ApoM may bind to LPS potentially facilitating endotoxin clearance by HDL. To this end, computational docking simulations were performed to predict the potential binding between ApoM and *E. coli* LPS.

**Fig. 2:**
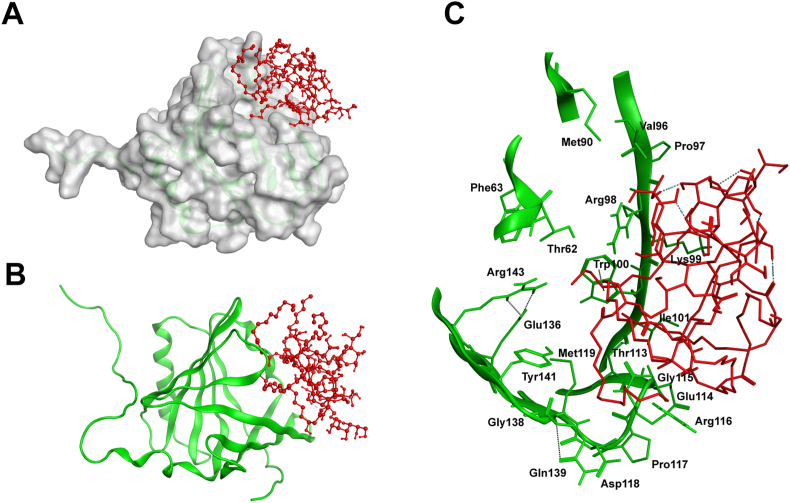
Potential interaction between ApoM and *E.coli* LPS revealed by docking simulations. **A**: Top-ranked model of ApoM-*E coli* LPS complex; ApoM is shown as a grey van der Waals surface, while *E. coli* LPS is presented as a red ball-and-stick model. **B**: Top-ranked model with ApoM in ribbon representation (green) and *E. coli* LPS shown as a red ball-and-stick model. **C**: A closer view of the ApoM-*E. coli* LPS binding interface. (For interpretation of the references to colour in this figure legend, the reader is referred to the Web version of this article.)

The best scoring model showed that *E. coli* LPS docks on the surface near the calyx of ApoM but does not block the S1P binding site inside the calyx (Fig. 2). The simulations results suggest a very strong binding, with Δ_r_G = - 41.46 kJ/mol (*K*_d_ = 52.8 nM) and root mean square deviation RMSD = 2.8 Å for the protein backbone C-α atoms. The interface binding sites between ApoM and *E. coli* is shown in Fig. 2C (more details can be found in the supplementary information accompanying the article).

### 3.3 Isothermal titration calorimetry study of the ApoM - *E. coli* LPS interaction

**Fig. 3:**
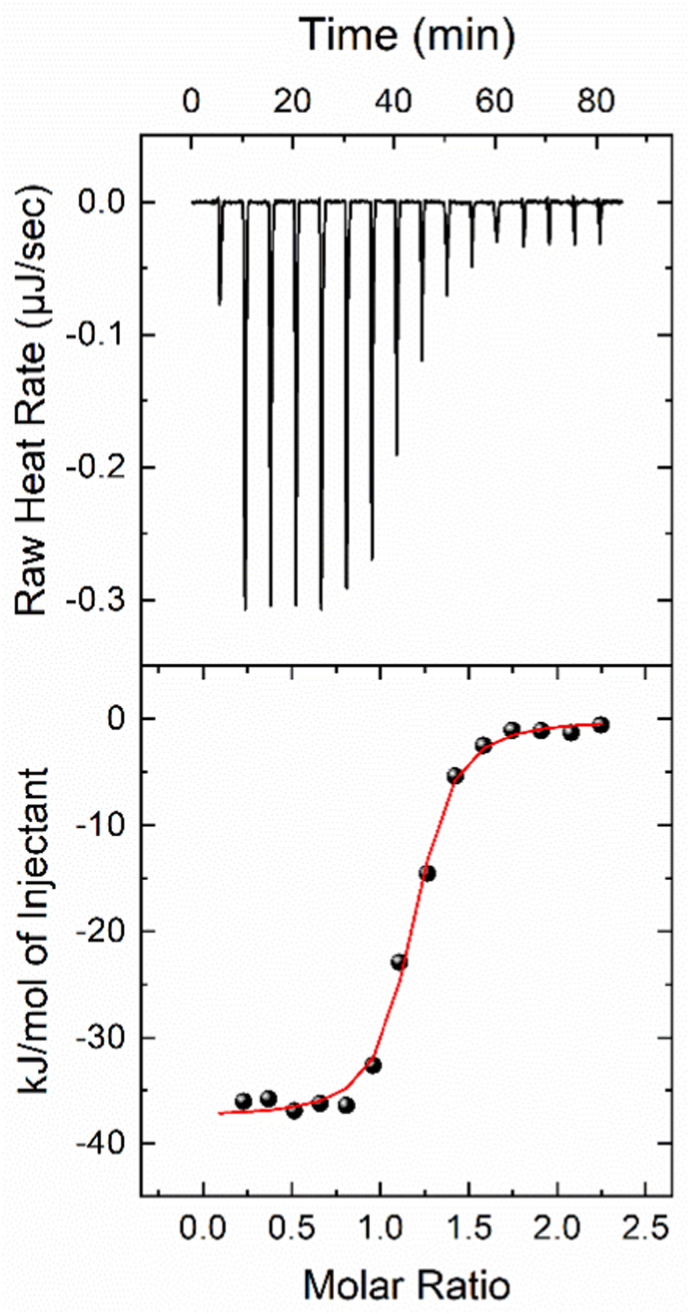
ITC measurements of ApoM and *E. coli* LPS interactions. (Upper Panel) Change of power supply to the calorimetric cell during the titration of 100 μM of LPS solution into 10 μM of ApoM in PBS buffer at 25 °C, after the subtraction of the appropriate reference experiments. Negative values indicate exothermic peaks. (Lower Panel) Integration of the area under each injection, normalized per mol of injectant and plotted as a function of the [LPS]/[ApoM] ratio at each point of the titration. Solid red line represent the non-linear least-square fit of the ITC data to a single-set-of-sites binding model. (For interpretation of the references to colour in this figure legend, the reader is referred to the Web version of this article.)

ITC was used to confirm the molecular simulations results and study the interaction between LPS and ApoM in more detail. The titration was performed at T = 25 °C, in PBS buffer and the ITC data are shown in Fig. 3. ApoM shows high affinity for *E. coli* LPS, forming 1:1 complex with a dissociation constant below 1 μΜ (Table 1), similar to the predicted *in silico* values (137 nM and 52 nM respectively). The binding process is strongly exothermic (Δ_r_H = −36.5 kJ/mol) and enthalpy-driven, implying the formation of an extensive network of interactions between ApoM and LPS when bound.

**Table 1:** Thermodynamic profile of the ApoM - *E. coli* LPS interaction, as determined by ITC at 25 °C.

Dissociation Constant [*K*_d_] (nM)	Stoichiometry	Binding Enthalpy [ΔrH]	Entropic Term	Gibbs Free Energy Change [ΔrG]
	[N]	(kJ/mol)	[-T∙ΔrS]	(kJ/mol)
			(kJ/mol)	
137.0 ± 3.4	1.06 ± 0.02	−36.5 ± 0.8	−2.7 ± 1.0	−39.2 ± 0.6

Table 1 footnotes: Dissociation constant [*K*_d_], binding enthalpy change [Δ_r_H], entropic term change [-T∙Δ_r_S] and free energy change [Δ_r_G] for the interaction between LPS and ApoM at T = 25 °C, in PBS buffer. Values and corresponding errors were derived from non-linear least square fit of the ITC data to a one-set-of-sites binding model and error propagation calculations.

### 3.4 ApoM neutralized *E. coli* LPS immune stimulatory activity *in vitro*

**Fig. 4:**
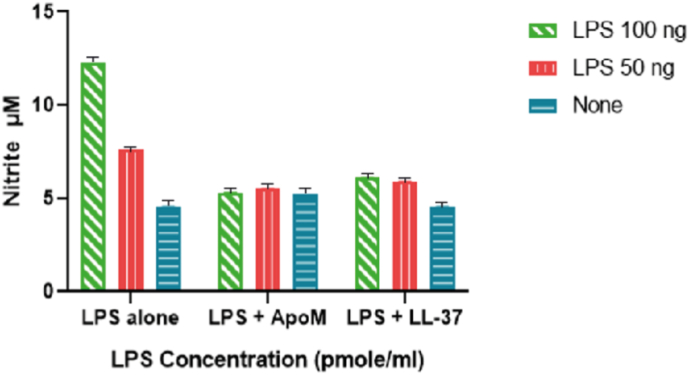
ApoM neutralized *E. coli* LPS and reduced nitric oxide release from macrophages *in vitro*. Freshly grown RAW264 macrophages were transferred to 96-well plate (0.25 × 10^6^ cell/well) then induced with *E. coli* LPS (100 and 50 ng/ml) pre-incubated with or without ApoM (10 μg/ml) for 30 min. LL37 was used as a control due to its ability to neutralize endotoxin. Plates were incubated overnight and nitrite accumulation was measured using the Greiss reaction method.

**Fig. 5:**
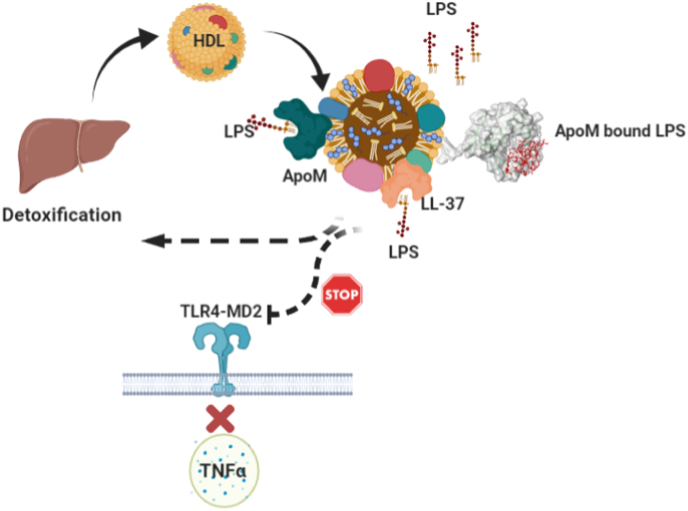
Schematic illustration of the interaction between LPS and the HDL-associated protein ApoM. LPS binds to ApoM surface near the calyx opening on the HDL particle. The formed complex is shuttled to liver for detoxification, therefore contributing to endotoxin clearance that reduced circulating LPS binding to TLR4-MD2. This process prevents the further activation of TLR4-MD2 and this prevents TNF-α overproduction.

To examine ApoM ability to neutralize endotoxin immune stimulatory activity, RAW264 macrophages were employed. Recombinant ApoM (10 μg/ml) where pre-incubated with *E. coli* LPS for 30 min then used to induced RAW264 macrophages. The data suggest that ApoM neutralized *E. coli* LPS biological activity and inhibited nitric oxide release from RAW264 macrophages (Fig. 4). Moreover, ApoM neutralizing effect was similar to LL-37 (Fig. 5) which was used as a control due to its endotoxin neutralizing activity [33]. Taken together, the data in this study demonstrate for the first time that ApoM binds to LPS with high affinity potentially contributing to endotoxin neutralization and clearance by HDL.

## 4 Discussion

HDL is known to confer anti-inflammatory effects in various conditions. For example, HDL inhibits atherogenesis formation consequently preventing cardiovascular disease progression [34,35]. Additionally, HDL modulate innate immunity and shown to minimize the immunological cellular responses to endotoxins by inhibiting the release of inflammatory mediators such as type I interferon [12,36]. Our data confirms that HDL neutralized endotoxins’ immune stimulatory activity and inhibited TNF-α release from human macrophage-like monocytic cells THP-1 hence HDL possessed anti-inflammatory properties. In support, Levine et al. investigated HDL levels in mice during endotoxemia and reported that higher HDL levels were associated with decreased plasma cytokines levels. Notably, the protective effect was observed in HDL transgenic mice, and upon intravenous infusion of reconstituted HDL [12].

The evidence for HDL anti-inflammatory properties is well established where alteration in HDL levels and compositions affects its functionality and protective properties [37]. Clinical trials in human showed that low HDL level was found to increase the immune system activation in response to low dose of LPS in healthy individuals [38]. In fact, HDL-associated proteins as ApoA1 was documented to bind endotoxin and, therefore, contributed to its neutralization [39]. Although ApoA1 is the main lipoprotein among HDL-associated proteins, others such as ApoC, ApoE, ApoL, ApoM, ApoJ, SAA, PON1 and 2AM play a role in HDL remodeling and functionality including its anti-inflammatory potential. Herein, we provide evidence that ApoM binds to endotoxin and plays a critical role in augmenting HDL anti-inflammatory activity.

ApoM is found associated mainly with HDL and to some lesser extent to LDL [40]. Ninety five percent of ApoM in circulation are bound to HDL [40]. ApoM is produced mainly in liver and kidney but the terminal signal peptide of ApoM is not cleaved prior releasing to plasma which helps ApoM in anchoring to HDL particles [41]. ApoM is a lipid carrier protein and member of the lipocalin protein family [42]. Lipocalins have a similar structure, consisting of 8 antiparallel β-strands, forming a β-barrel fold (calyx) that protects an internal binding site [43]. ApoM structure resembles that of many other lipocalins, including MD2, the co-receptor for TLR4. MD2 is composed of 2 β-strands forming a cup-like structure [42]. The high structural similarity of ApoM and MD2 folds suggests the ability to bind endotoxins. Our docking simulation studies resulted a high-scoring ApoM -*E. coli* LPS complex that we used to gain further insight into this interaction. Based on this model, ApoM binds LPS on the surface near the calyx opening but does not mask the binding site pocket where S1P ligand is shuttled. Further investigations using ITC confirmed this interaction. A high affinity binding between ApoM and *E. coli* LPS was confirmed forming 1:1 complexes with *K*_d_ values below 1 μΜ. The binding process is strongly exothermic and enthalpy-driven (ΔrH = −36.5 kJ/mol), implying the formation of an extensive network of interactions between ApoM and LPS in the bound state. Our experiments were performed to mimic the physiological role of circulating ApoM, which is usually bound to HDL in endotoxin clearance. Our novel data suggests that ApoM contributes to endotoxin neutralization and clearance by HDL.

Functionally, ApoM is the carrier of sphingosine-1-phosphate (S1P), a bioactive lipid mediator that modulates vascular inflammation. Some studies documented that ApoM - S1P complex is important for HDL antiatherogenic and anti-inflammatory effect [44]. ApoM – S1P complexes are upregulated during inflammatory status [45]. However, Winkler et al. study reported a decline in S1P level in septic shock patients and suggested that the drastic decrease of HDL level during septic shock is the reason for this decline [46]. Our computational docking simulation showed that *E.coli* LPS does not occupy the binding site of S1P in internal section of the calyx, however it is not known if the ApoM bind both of LPS and S1P simultaneously which warrants further investigation.

In this study, we showed that ApoM neutralized endotoxin activity and thereby decreased nitric oxide production in murine RAW264 macrophages. Endotoxins recognition or sensing is mediated by TLR4-MD2 receptor complex, mainly by the extracellular domain of TLR4 [47]. Upon LPS binding to MD2 co-receptor of TLR4, the dimerization of TLR4 ectodomains occur leading to conformational changes that initiate signal transduction through MYD88 and TRIFF signaling pathways leading to inflammatory mediators’ release [29]. The signal stimulates the production of the acute phase reactants such as SAA, TNF-α, IL-6 [48]. Based on our data, we suggest that the binding of LPS to HDL-associated protein ApoM facilitates shuttling endotoxins to liver for detoxification. The later process prevents the activation of TLR4, consequently suppressing the stimulation of downstream pathways leading to reduced production of pro-inflammatory mediators and the acute phase reactants (Fig. 5).

In support of our conclusion, we used LL-37 to inhibit LPS immune stimulatory activity. LL-37 or cathelicidin or hCAP18 plays a similar role in inhibiting the interaction between LPS and LPS-binding protein, consequently preventing TLR4 activation and reduced TNF-α release [[49], [50], [51]]. Furthermore, LL-37 found to suppress inflammation and cell death via inhibiting the IL-1β expression and caspase-1 activation [52]. Therefore, we used LL-37 as a control to study the ApoM interaction with LPS and provide a supporting evidence to its role in potentiating the anti-inflammatory activity of HDL.

## 5 Conclusion

ApoM binds with high affinity to *E coli* LPS to facilitate endotoxin neutralization and clearance by HDL.

## Author contributions

Conceived the study: S.M.Z.; data collection: S.M.Z, AT, and H.M.; data analysis: H.M., AT, and S.M.Z. Supervision: S.M.Z. Writing manuscript draft: H.M., AT, S.M.Z. Critically reviewing and finalizing manuscript S.M.Z, AT, H.M. All authors have read and agreed to the published version of the manuscript.

## Funding

This work is funded by a graduate student grant QUST-2-CMED-2019-6 from 10.13039/501100004252Qatar University. This work in part is funded by Qatar National research Fund (NPRP12S-0224–190144) to SMZ.

## Declaration of competing interest

All authors declare no conflict of interests.

## Data Availability

No data was used for the research described in the article.
